# Hydrogen Sulfide Inhibits Cigarette Smoke-Induced Endoplasmic Reticulum Stress and Apoptosis in Bronchial Epithelial Cells

**DOI:** 10.3389/fphar.2017.00675

**Published:** 2017-09-28

**Authors:** Fan Lin, Chengcheng Liao, Yun Sun, Jinsheng Zhang, Weiwei Lu, Yu Bai, Yixuan Liao, Minxia Li, Xianqiang Ni, Yuelong Hou, Yongfen Qi, Yahong Chen

**Affiliations:** ^1^Department of Respiratory and Critical Care Medicine, Peking University Third Hospital, Beijing, China; ^2^Department of Respiratory Medicine, Zhejiang Provincial People's Hospital, Hangzhou, China; ^3^Key Laboratory of Molecular Cardiovascular Science, Ministry of Education, Peking University Health Science Center, Beijing, China

**Keywords:** hydrogen sulfide, chronic obstructive pulmonary disease, bronchial epithelial cell, apoptosis, endoplasmic reticulum stress

## Abstract

**Background:** Apoptosis of lung structural cells contributes to the process of lung damage and remodeling in chronic obstructive pulmonary disease (COPD). Our previous studies demonstrated that exogenous hydrogen sulfide (H_2_S) can reduce the lung tissue pathology score, anti-inflammation and anti-oxidation effects in COPD, but the effect of H_2_S in regulating cigarette smoke (CS) induced bronchial epithelial cell apoptosis and the underlying mechanisms are not clear.

**Objectives:** To investigate the effect of H_2_S on CS induced endoplasmic reticulum stress (ERS) and bronchial epithelial cell apoptosis.

**Methods:** Male Sprague–Dawley rats randomly divided into four groups for treatment: control, CS, NaHS + CS, and propargylglycine (PPG) + CS. The rats in the CS group were exposed to CS generated from 20 commercial unfiltered cigarettes for 4 h/day, 7 days/week for 4 months. Since the beginning of the third month, freshly prepared NaHS (14 μmol/kg) and PPG (37.5 mg/kg) were intraperitoneally administered 30 min before CS-exposure in the NaHS and PPG groups. 16HBE cells were pretreated with Taurine (10 mM), 5 mmol/L 4-phenylbutyric acid (4-PBA) or NaHS (100, 200, and 400 μM) for 30 min, and then cells were exposed to 40 μmol/L nicotine for 72 h. ERS markers (GRP94, GRP78) and ERS-mediated apoptosis markers 4-C/EBP homologous protein (CHOP), caspase-3 and caspase-12 were assessed in rat lung tissues and human bronchial epithelial cells. The apoptotic bronchial epithelial cells were detected by Hoechst staining *in vitro* and TUNEL staining *in vivo*.

**Results:** In CS exposed rats, peritoneal injection of NaHS significantly inhibited CS induced overexpression ERS-mediated apoptosis markers and upregulation of apoptotic rate in rat lungs, and inhibiting the endogenous H2S production by peritoneal injection of PPG exacerbated these effects. In the nicotine-exposed bronchial epithelial cells, appropriate concentration of NaHS and ERS inhibitors taurine and 4-PBA inhibited nicotine-induced upregulation of apoptotic rate and overexpression of ERS-mediated apoptosis markers.

**Conclusion:** H_2_S inhibited lung tissue damage by attenuating CS induced ERS in rat lung and exogenous H_2_S attenuated nicotine induced ERS-mediated apoptosis in bronchial epithelial cells.

## Key messages

**What is the key question?**

Does hydrogen sulfide (H_2_S) inhibits bronchial epithelial cell apoptosis, and if so, do endogenous H_2_S inhibits bronchial epithelial cell apoptosis through regulating endoplasmic reticulum stress (ERS)?

**What is the bottom line?**

This study is the first to demonstrate that H_2_S can inhibit cigarette smoke induced ERS mediated apoptosis, especially in bronchial epithelial cells.

**Why read on?**

As cigarette smoke is an independent risk factor for chronic obstructive pulmonary disease (COPD) and ERS is a crucial process in the pathogenesis of COPD, our findings suggest that H_2_S has potential therapeutic value in the treatment of respiratory diseases such as COPD.

## Introduction

Chronic obstructive pulmonary disease (COPD) is characterized by persistent airflow limitation that is usually progressive and associated with an enhanced chronic inflammatory response in the airways and the lung to noxious particles or gases. Apoptosis of lung structural cells, the proteinase-antiproteinase hypothesis, oxidant-antioxidant balance, inflammation and immunity are believed to contribute to the process of lung damage and remodeling in COPD (Chung and Adcock, 2008; Vijayan, 2013). Emerging evidence indicates that endoplasmic reticulum stress (ERS), which is characterized by an abnormal formation of inclusion bodies and aggregation of misfolded proteins (Min et al., 2011; Ribeiro and O'Neal, 2012; Wei et al., 2013) may play an important role in the development or pathology of COPD. In endoplasmic reticulum, glucose regulated protein 78 (GRP78) binds to transmembrane sensor protein PKR-like endoplasmic reticulum Kinase (PERK), activating transcription factor 6 (ATF6), and inositol-requiring enzyme 1 (IRE1), maintaining each in its inactive state (Osorio et al., 2013). During ERS, GRP78 interacts with these nascent proteins and is released from these transmembrane sensors, these proteins then act to maintain homeostasis and normal functioning (Tanjore et al., 2012; Marciniak, 2017). However, when these mechanisms fail, apoptotic cell death can occur (Lin et al., 2008). Apoptosis of lung structural cells caused by ERS-mediated apoptosis may play a role in the pathogenesis of COPD. Min (Min et al., 2011) demonstrated the expression of ERS-mediated apoptosis marker increased in the lungs of COPD patients. And cigarette smoke (CS) can induce ERS and ERS-mediated apoptosis in the human lung cells (Kelsen et al., 2008; Geraghty et al., 2011; Somborac-Bacura et al., 2013). Therefore, it is logical to speculate that inhibiting CS-induced ERS may be a novel therapeutic strategy to prevent and ameliorate apoptosis and lung damage.

Hydrogen sulfide (H_2_S) is commonly considered as an environmental contaminant. However, studies show that H_2_S plays a pivotal role as an important endogenous modulator (Paul and Snyder, 2015; Szabo, 2017). In the respiratory tract, endogenous H_2_S has been shown to participate in the regulation of important functions such as airway tone, pulmonary circulation, cell proliferation or apoptosis, fibrosis, oxidative stress, inflammation and antiviral defenses (Bazhanov et al., 2017). Our previous studies demonstrated that exogenous H_2_S can degrade airway reactivity, reduce the lung tissue pathology score, anti-inflammation and anti-oxidation effects in COPD (Chen et al., 2011). However, the mechanisms underlying the protective effect of H_2_S against COPD are not completely understood. Recent studies have demonstrated that H_2_S inhibits ERS-mediated apoptosis in many organs. Wei et al. (2014) indicates that H_2_S inhibits homocysteine-induced ERS and neuronal apoptosis in rat hippocampus. Li et al. (2015) found H_2_S protects against myocardial ischemia/reperfusion injury in rats by inhibiting ERS. In addition, H_2_S can attenuate 6-hydroxydopamine-induced ERS in SH-SY5Y cell line (Xie et al., 2012). And H_2_S decreased the expression of ERS-mediated apoptosis marker 4-C/EBP homologous protein (CHOP) and cardiomyocytic injury induced by hyperhomocysteinemia (Wei et al., 2010).

We therefore assess ERS and apoptosis markers in lung tissues from chronic CS exposed rats and nicotine-exposed bronchial epithelial cell model, and investigate whether H_2_S can suppress bronchial epithelial cell apoptosis through regulating ERS.

## Materials and methods

### Animal model

All procedure relating to animal care and treatments strictly adhered to the ethical procedures and policies approved by Animal Care and Use Committee of National Research Center.

Male Sprague–Dawley rats weighing 200–250 g were supplied by the Animal Center, Health Science Center, Peking University, and randomly divided into four groups (each *n* = 8) for treatment: control, CS, Sodium hydrosulfide (NaHS, a H_2_S donor) + CS and propargylglycine (PPG, which is a cystathionine γ-lyase inhibitor can inhibit the endogenous H_2_S production) + CS. The rats in the CS group were exposed to whole-body mainstream CS generated from 20 commercial unfiltered cigarettes in a dynamic smoke exposure box for 4 h/day, 7 days/week. Since the beginning of the third month, freshly prepared NaHS (14 μmol/kg) and PPG (37.5 mg/kg) were intraperitoneally administered 30 min before CS-exposure in the NaHS and PPG groups. Rats were anesthetized by intraperitoneal injection of 20% (w/v) urethane (5 mL/kg) 24 h after 4-month exposure.

### 16HBE cell culture and treatment

The human bronchial epithelial cell line 16HBE was purchased from Shanghai Bogoo Biotechnology.Co., Ltd. 16HBE cells were maintained in complete growth medium (RPMI 1640) supplemented with 10% fetal bovine serum (FBS, Gibco), 2 mM L-glutamine, 100 U/ml penicillin, and 100 mg/ml streptomycin at 37°C in a humidified atmosphere with 5% CO_2_. Different drugs were tested on the 16HBE cells. The Taurine, 4-phenylbutyric acid (4-PBA) and NaHS were dissolved in PBS, nicotine (5, 10, 20, 40, and 80 μM) was prepared in DMSO. DMSO was added to the control and to the samples when necessary. 16HBE cells were pretreated with Taurine (10 mM), 4-PBA(5 mM) or NaHS (100, 200, and 400 μM) for 30 min, and then cells were exposed to 40 μmol/L nicotine for 72 h.

### Hoechst staining assay

16HBE cells were cultured in 6-well cell culture plates, media was removed from the wells and the scaffolds along with cells were washed twice with PBS solution and fixed with 2.5% glutaraldehyde overnight at −4°C. Cells were then stained with 10 μg/ml of Hoechst 33342. Changes in morphology were detected by fluorescence microscopy using a filter for Hoechst 33342 (365 nm). After Hoechst staining, apoptotic nuclei appear condensed (pyknotic) and bright blue (Woo, 1995). For quantification of Hoechst 33342 staining, the percentage of apoptotic nuclei per optical field (at least 50 fields) was counted.

### TUNEL staining assay

The apoptotic cells in rat lung were identified by TUNEL staining (Roche Applied Science). Briefly, deparaffinized and fixed sections were immersed in 20 mg/ml proteinase K for 15 min. After refixation and equilibration, sections were incubated with 50 μl of TUNEL reaction mixture at 37°C for 1 h. Sections were also stained with Hoechst. Images were viewed under an inverted microscope (Leica DMI3000B, Wetzlar, Germany). The airways of 2 mm and less in diameter were pictured. For apoptotic cells, we examined positive fluorescent apoptotic nuclei in 20 low power fields/slide at 10 × magnification 40 fields per section were randomly manually selected by a single observer who was blinded to the intervention.

### Western blot analysis

Protein extracts from lung tissues and the 16HBE cells were resolved by 10% SDS-PAGE and then transferred to a nitrocellulose membrane. Then the nitrocellulose membrane was incubated with the primary antibodies anti-CHOP (1:1000), anti-GRP94 (1:3000), anti-GRP78 (1:3000), anti-cleaved caspase12 (1:500), anti-precaspase12 (1:500) or anti-β-actin (1:3000) overnight, then secondary antibody (horseradish peroxidase-conjugated anti-goat or anti-rabbit IgG) for 1 h. The reaction was visualized by enhanced chemiluminescence. Protein contents were normalized to that of β-actin or glyceraldehyde-3-phosphate dehydrogenase (GAPDH).

### Hematoxylin and eosin (HE) staining

Rat lungs were excised and fixed, embedded and cut into 5 μm thick sections, stained with hematoxylin for 15 min and with eosin for 3 min, underwent ethanol dehydration, xylene transparency, and neutral gum mounting, and observed under a microscope.

### Statistical analysis

Graphpad software (GraphPad Prism v5.00 for Windows; GraphPad Software Inc., San Diego, CA, USA) was used for analyzing data, which were expressed as mean ± SD. Comparisons among more than 2 groups were analyzed by one-way analysis of variance followed by Student–Newman–Keuls test. *P* < 0.05 was considered statistically significant.

## Results

### H_2_S alleviated Cs induced lung tissue damage and cell apoptosis

**Figure 1:**
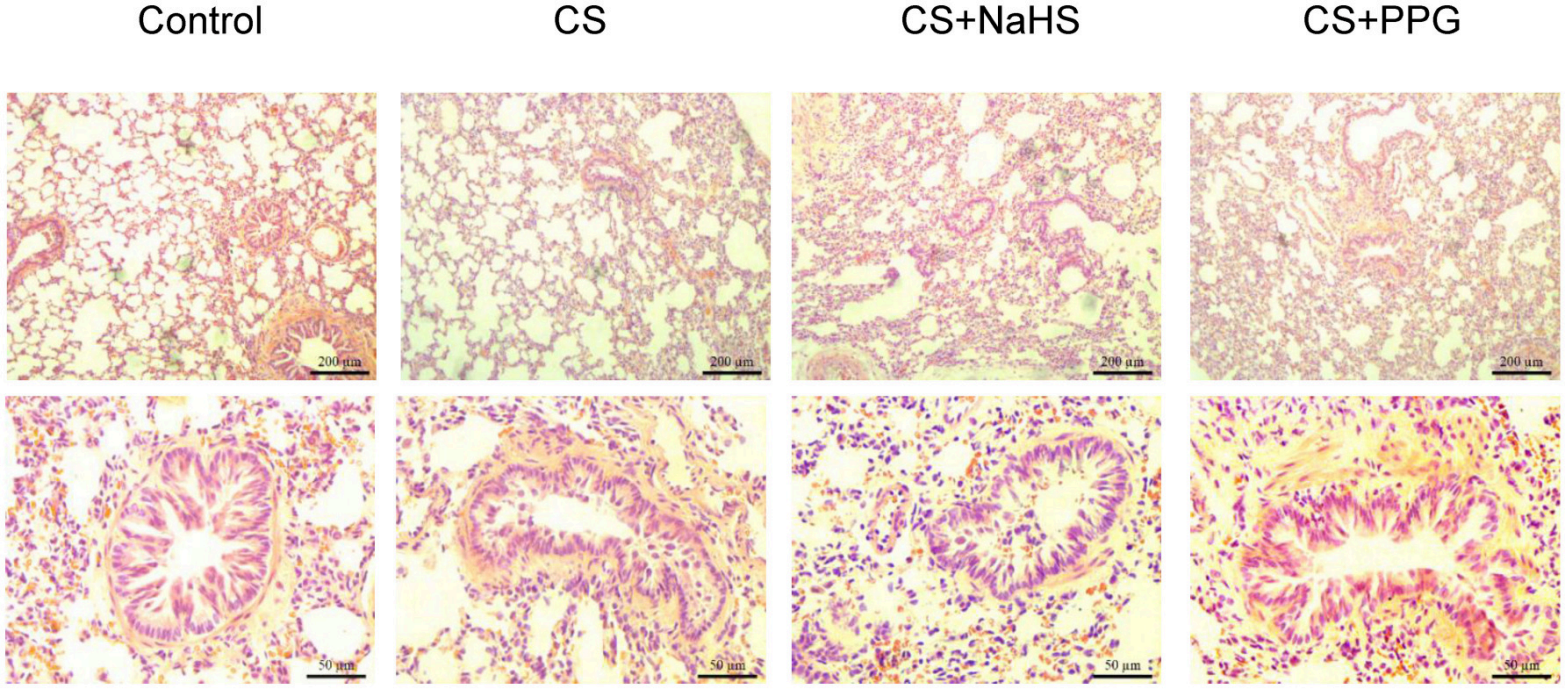
Changes in lung pathology and small airway fibrosis in rats. Lung tissue were stained with hematoxylin and eosin and examined on light microscopy. HE × 100, × 400.

HE staining showed inflammatory cells infiltration, destruction of alveolar walls and enlargement of airspaces were observed in lung tissue of passive smoking rats. Peritoneal injection of NaHS significantly reduced these lung pathological damages compared with CS alone. Meanwhile, peritoneal injection of PPG exacerbated these lung pathological damages compared with CS alone (Figure 1).

**Figure 2:**
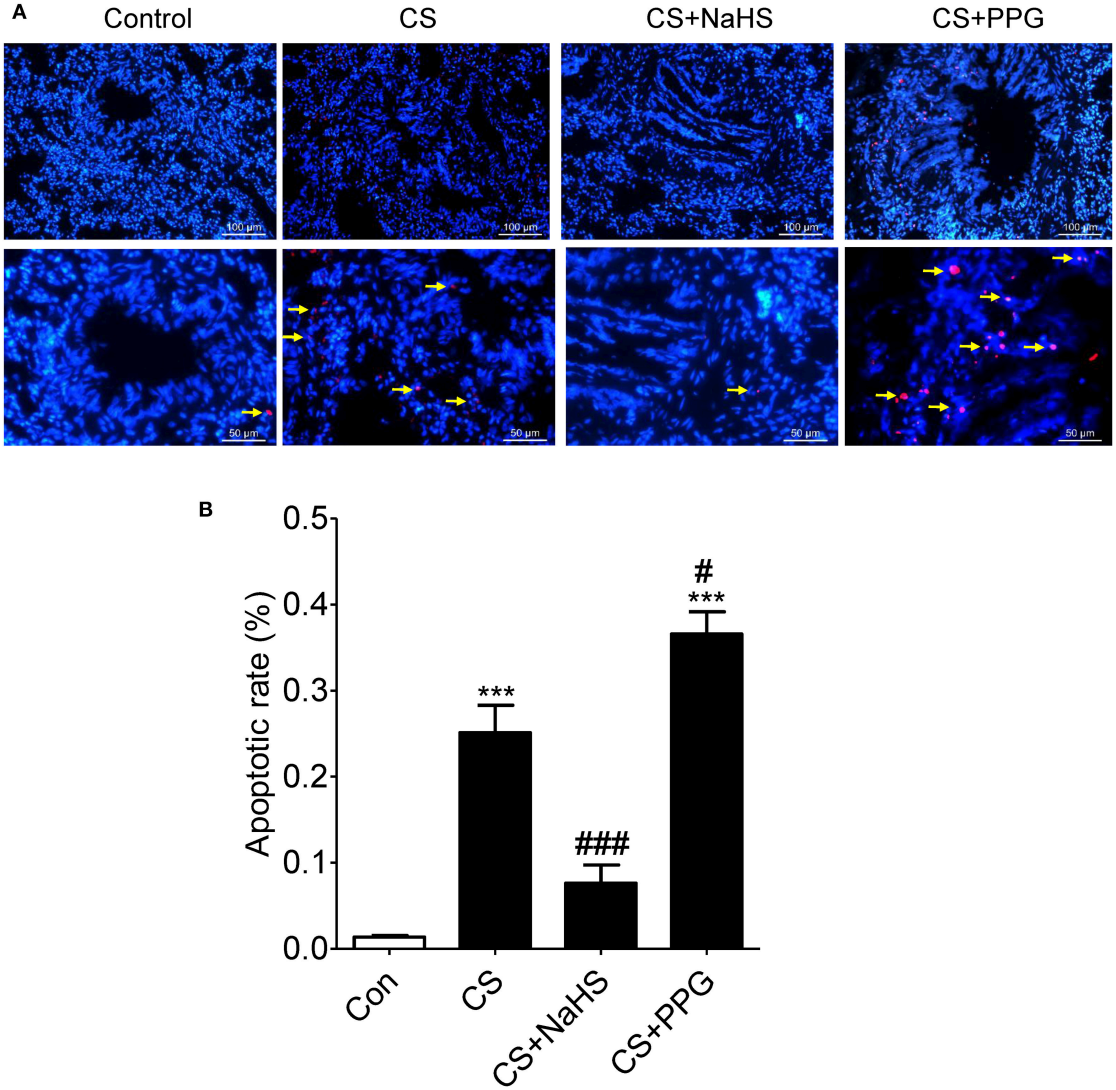
Endogenous H_2_S alleviated CS induced lung structure cell apoptosis. **(A)** Red: TUNEL-positive nuclei; Blue: Hoechst33258 which stained nucleus and the colocalization (white arrow) was shown with TUNEL at right. The airways of 2 mm and less in diameter were pictured. All Hoechst-positive nuclei as well as TUNEL-positive nuclei were visualized under an inverted microscope (Leica DMI3000B, Wetzlar, Germany). **(B)** The number of colocalization nuclei was counted in 10 random high-power (HP) fields in at least 6 lung tissue slides in each group. Values are expressed as mean ± SEM. ^***^P < 0.001 vs. control group and ^#^*P* < 0.05 and ^###^*P* < 0.001 vs. CS group.

Compared with control group, apoptotic rate (the percent of the number of colocalization nuclei) was increased by 1,754.1% (*P* < 0.0001) in the CS group and increased by 2601.2% (*P* < 0.0001) in the PPG group. Compared with the CS group, apoptotic rate was decreased by 69.6% (*P* < 0.001) in the NaHS group. On the contrary, compared with the CS group, apoptotic rate was increased by 45.7% (*P* < 0.05) in the PPG group (Figures 2A,B).

### H_2_S inhibits ERS and ERS-mediated apoptosis in rat lung induced by CS

**Figure 3:**
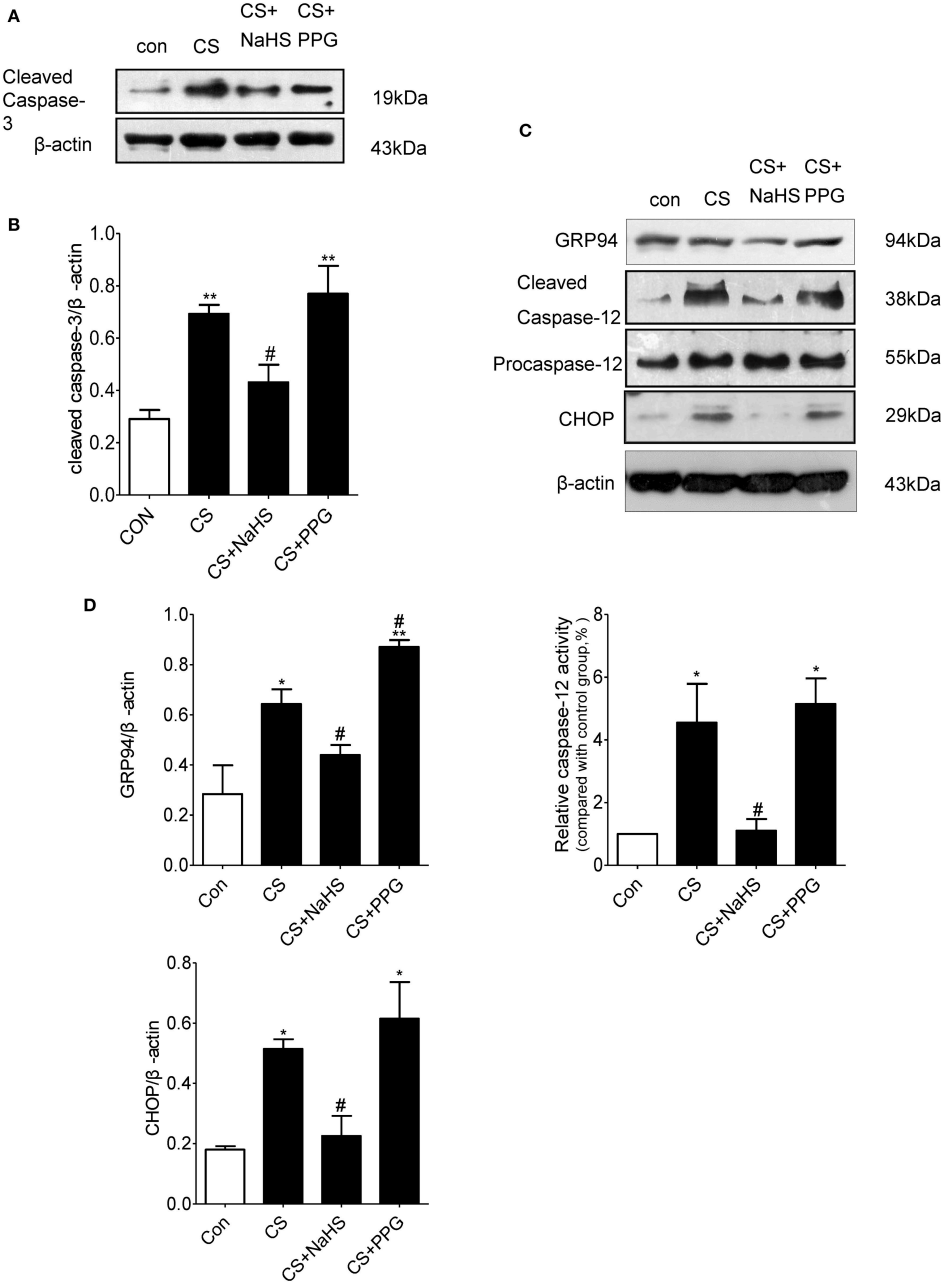
Endogenous H_2_S inhibits ERS-mediated apoptosis in rat lung induced by CS. **(A,B)** Changes in cleaved caspase-3 protein expression levels in the lung homogenates of rats. **(C,D)** Changes in GRP94, CHOP and cleaved caspase-12 protein expression levels in the lung homogenates of rats. Results are presented for *n* = 8 mice per group and of 3 independent experiments (*n* = 3). Values are expressed as mean ± SEM. ^*^P < 0.05, ^**^P < 0.01 vs. control group and ^#^*P* < 0.05 vs. CS group.

Western blot results showed that the expression of apoptosis marker cleaved caspase-3 in the lung tissues of CS group increased by 138.3% (*P* < 0.01) compared with control group. Compared with CS group, the expression of cleaved caspase-3 in the lung tissues of NaHS group decreased by 37.7% (*P* < 0.05), and the expression of cleaved caspase-3 in the lung tissues of PPG group increased by 11.1% (*P* = 0.5313; Figures 3A,B).

Compared with control group, the expression of ERS markers GRP94 and apoptosis markers CHOP and cleaved caspase-12 in the lung tissues of CS group increased by 126.5, 186.2, and 355.1%, respectively (all *P* < 0.05). Compared with CS group, the expression of GRP94, CHOP, and cleaved caspase-12 in the lung tissues of NaHS group decreased by 45.8, 56.3, and 72.8%, respectively (all *P* < 0.05; Figures 3C,D).

### H_2_S inhibits nicotine-induced morphological changes of apoptosis in bronchial epithelial cell *in vitro*

**Figure 4:**
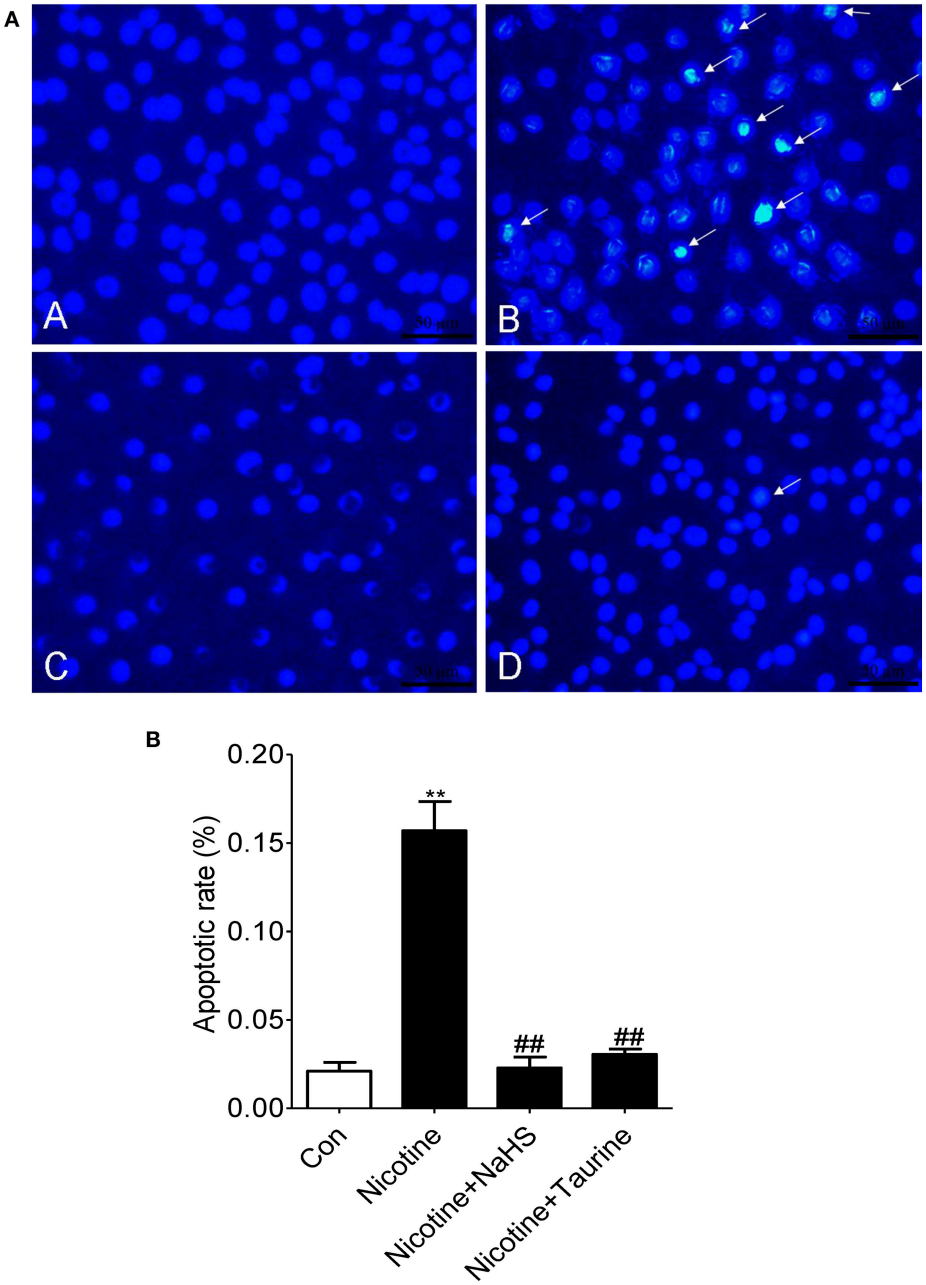
H_2_S inhibits nicotine-induced morphological changes of apoptosis in bronchial epithelial cell *in vitro*. **(A)** Hoechst 33258, a DNA-specific dye, was used to detect morphologic apoptosis in bronchial epithelial cells. After Hoechst staining, apoptotic nuclei appear condensed (pyknotic) and bright blue (×400). A: the control group; B: the 40 μmol/L nicotine group; C: the 200 μmol/L NaHS + 40 μmol/L nicotine group; D: the 10 mmol/L taurine + 40 μmol/L nicotine group. **(B)** The percentage of apoptotic nuclei (white arrow) per optical field was counted. Values are expressed as mean ± SEM. ^**^P < 0.01 vs. control group and ^##^*P* < 0.01 vs. nicotine group.

Hoechst staining results showed that an even and diffuse blue fluorescence appears in 16HBE cell nuclei in control group. Typical morphological changes of apoptosis such as a thick mass, high concentration fluorescence and the nucleus chromatin pieces appears in the 16HBE cells treated with 40 μmol/L nicotine for 72 h. Pre-exposure 16HBE cells to 200 μmol/L NaHS or 10 mmol/L taurine for 30 min significantly alleviated the apoptotic nuclei morphological changes, suggesting that NaHS may alleviate nicotine induced bronchial epithelial cell apoptosis (Figure 4A).

Compared with control group, apoptotic rate (the percent of Hoechst-positive nuclei) was increased by 647.1% (*P* < 0.01) in the nicotine group. Compared with the nicotine group, apoptotic rate was decreased by 85.4% (*P* < 0.01) in the NaHS + nicotine group, and apoptotic rate was decreased by 80.6% (*P* < 0.01) in the taurine + nicotine group (Figure 4B).

### H_2_S inhibits human bronchial epithelial cell apoptosis via regulating ERS *in vitro*

**Figure 5:**
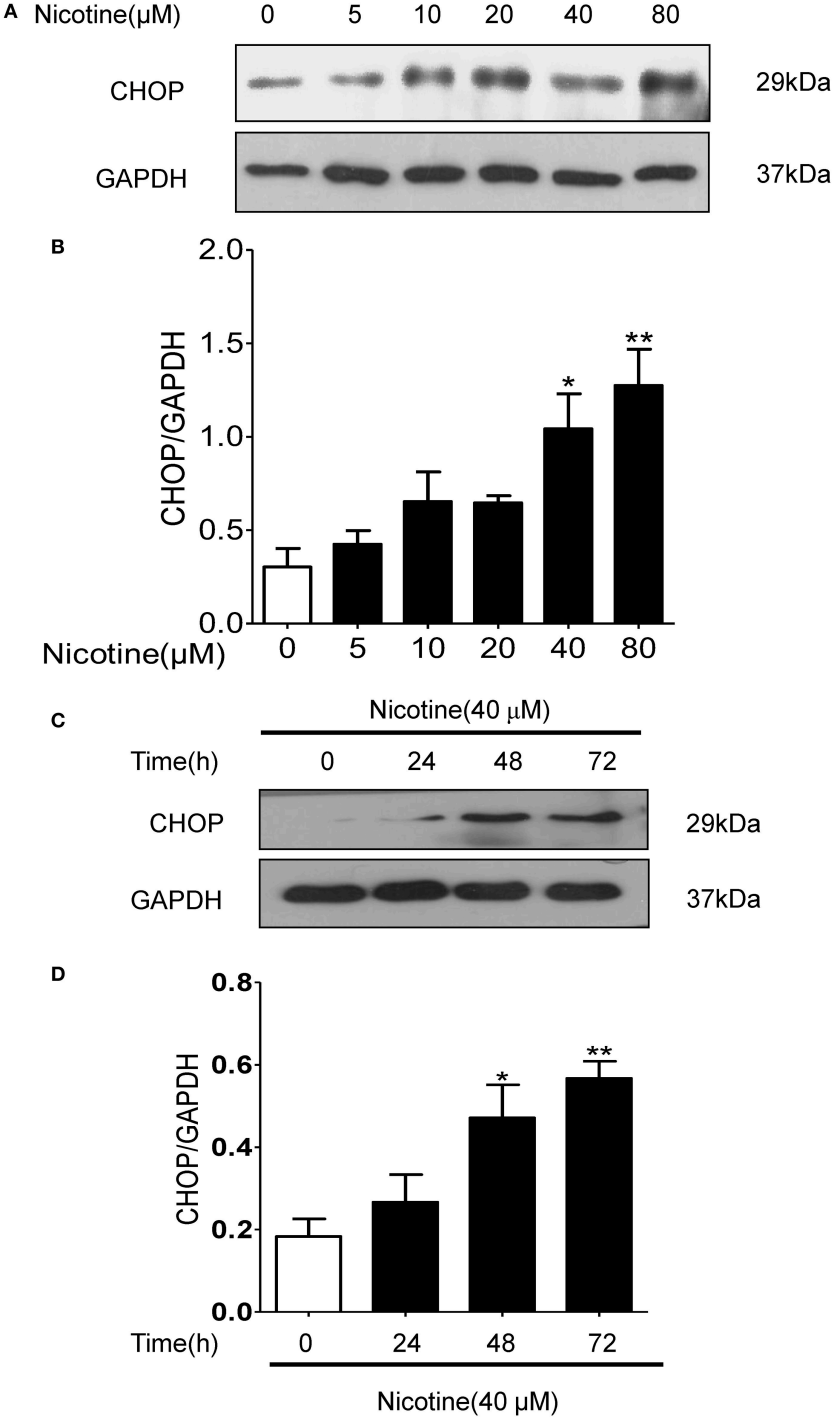
Nicotine concentration and time dependently increased the expression of CHOP in 16HBE cells. **(A,B)** With the increase of nicotine concentration, CHOP protein expression increased gradually. **(C,D)** With the increase of reaction time, CHOP protein expression increased gradually. Results are presented by 3 independent experiments (*n* = 3). Values are expressed as mean±SEM. ^*^P < 0.05, ^**^P < 0.01 vs. 0 μmol/L nicotine group.

Nicotine concentration and time dependently increased the expression of CHOP in 16HBE cells (Figures 5A–D). Compared with the control group, the expression of CHOP in the group with 40 μmol/L nicotine treated for 72 h significantly increased by 244.4% (*P* < 0.05; Figure 5D).

**Figure 6:**
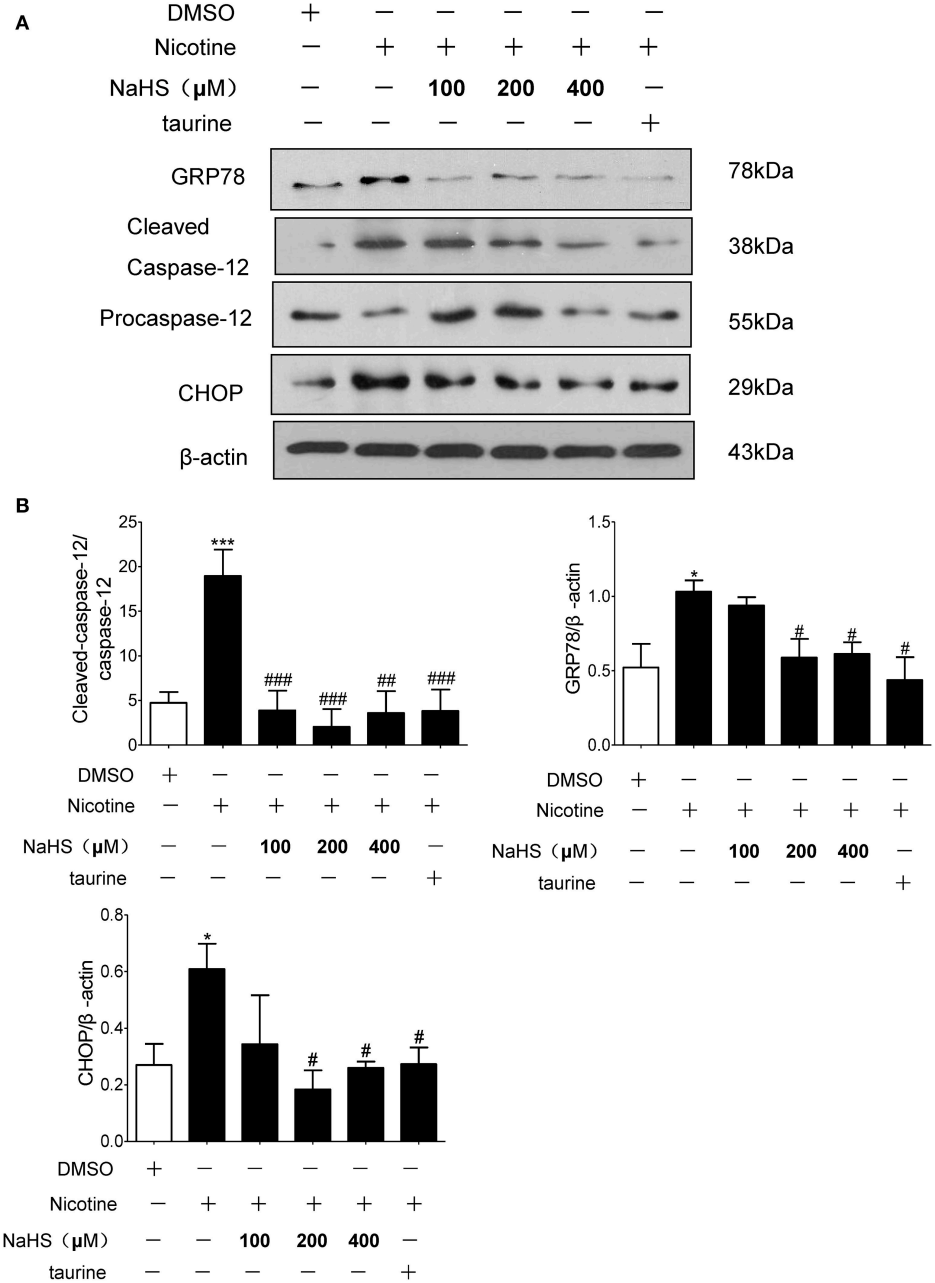
H_2_S and ERS inhibitor taurine suppressed nicotine induced ERS and ERS-mediated apoptosis in 16HBE cells. **(A)** 16HBE cells were pretreated with Taurine (10 mM) or NaHS (100, 200, and 400 μM) for 30 min, and then cells were exposed to 40 μmol/L nicotine for 72 h. **(B)** Results are presented of 3 independent experiments (*n* = 3). Values are expressed as mean ± SEM. ^*^P < 0.05, ^***^P < 0.001 vs. DMSO group. ^#^*P* < 0.05, ^##^*P* < 0.01, ^###^*P* < 0.001 vs. nicotine group.

**Figure 7:**
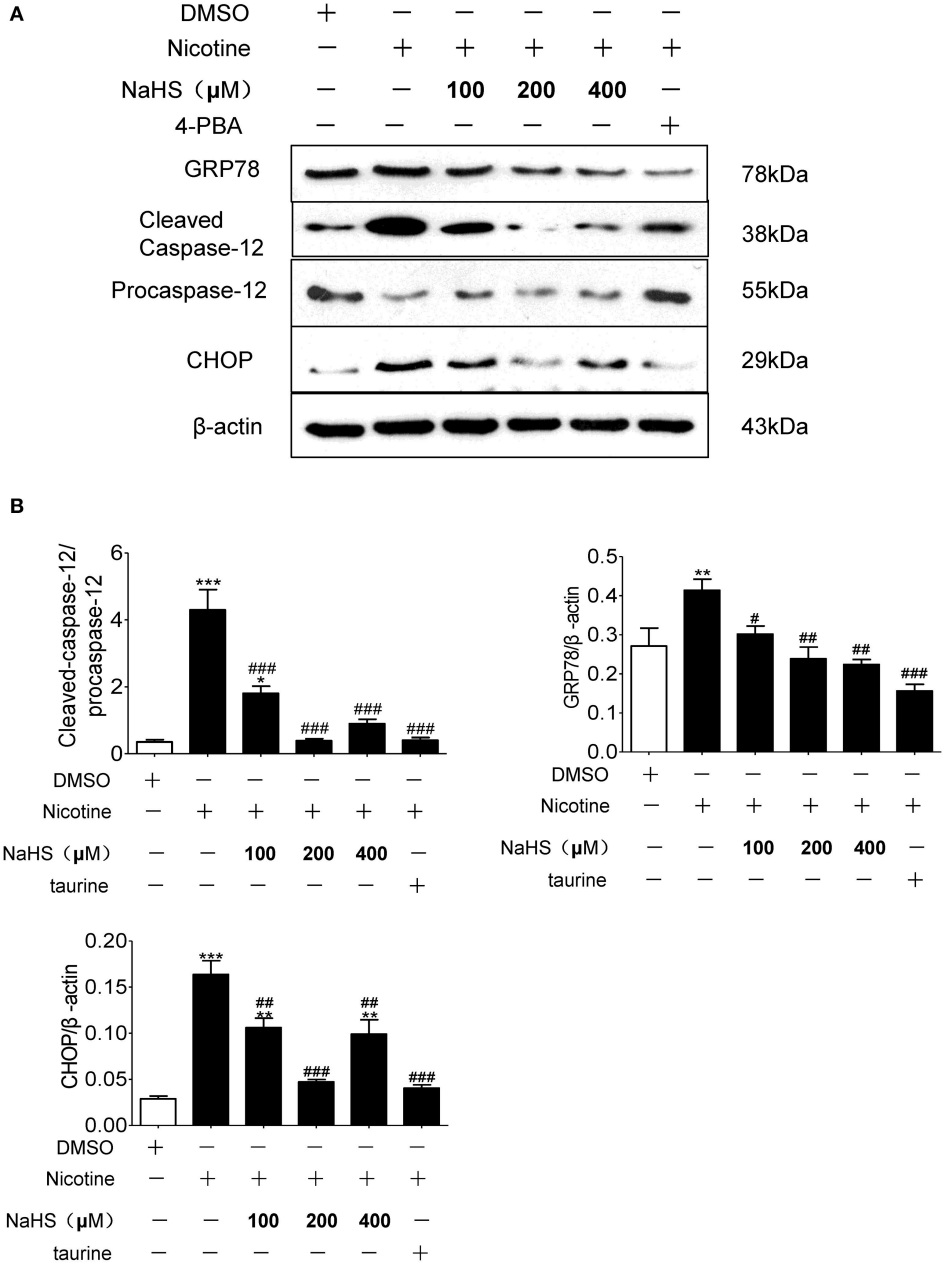
H_2_S and ERS inhibitor 4-PBA suppressed nicotine induced ERS and ERS-mediated apoptosis in 16HBE cells. **(A)** 16HBE cells were pretreated with 4-PBA (5 mM) or NaHS (100, 200, and 400 μM) for 30 min, and then cells were exposed to 40 μmol/L nicotine for 72 h. **(B)** Results are presented of 3 independent experiments (*n* = 3). Values are expressed as mean ± SEM. ^*^P < 0.05, ^**^P < 0.01, ^***^P < 0.001 vs. DMSO group. ^#^*P* < 0.05, ^##^*P* < 0.01, ^###^*P* < 0.001 vs. nicotine group.

Compared with the control group, the protein expression of GRP78, CHOP and cleaved caspase-12 in nicotine group increased by 106.2% (*P* < 0.05), 132.2% (*P* < 0.05), and 201.4% (*P* < 0.01). Compared with the 40 μmol/L nicotine group, the protein expression of GRP78, CHOP, and cleaved caspase-12 in the 200 μmol/L NaHS+ nicotine group were significantly decreased by 40.7% (*P* < 0.05), 66.2% (*P* < 0.05), and 99.8% (*P* < 0.01), respectively. In the 400 μmol/L NaHS+ nicotine group, they were decreased by 40.0% (*P* < 0.05), 56.3% (*P* < 0.05), and 92.3% (*P* < 0.01). In the taurine + nicotine group, they were decreased by 58.6% (*P* < 0.05), 56.3% (*P* < 0.05), and 80.4% (*P* < 0.01) (Figures 6A,B). Similarly, In the 4-PBA + nicotine group, they were decreased by 62.25, 71.18, and 90.91% (all *P* < 0.001; Figures 7A,B).

## Discussion

Our findings revealed that ERS was induced in the lung tissues of CS-exposed rats and endogenous H_2_S inhibited ERS and ERS-mediated apoptosis in the lung tissues of CS-exposed rats. H_2_S protected against nicotine-induced bronchial epithelial cell apoptosis *in vitro*, at least in part through regulating ERS. These findings may reveal the mechanism through which H_2_S protects against COPD.

Apoptosis is involved in the development of COPD and it has been demonstrated that there is an increase in apoptotic alveolar epithelial and endothelial cells in the lungs of COPD patients (Demedts et al., 2006). Since this is not counterbalanced by an increase in proliferation of these structural cells, the net result is destruction of lung tissue and the development of emphysema. Caspase-3, Bcl-2 family, vascular endothelial growth factor and so on have been defined to be involved in lung epithelial cell apoptosis, thus causing lung damage and emphysema (Aoshiba et al., 2003; Demedts et al., 2006; Mouded et al., 2009; Zeng et al., 2012). ERS-mediated apoptosis of lung structural cells may play a role in the pathogenesis of COPD. Min (Min et al., 2011) demonstrated the expression of ERS-mediated apoptosis markers increased in the lungs of subjects with COPD. Cigarette smoking as the most common risk factor of respiratory diseases has been confirmed to induce ERS and ERS-mediated apoptosis markers in the human lung cells (Kelsen et al., 2008; Geraghty et al., 2011; Somborac-Bacura et al., 2013). Most of the activity of the CS condensate was in basic fractions and in a weakly acidic fraction (Kier et al., 1974; Hutton and Hackney, 1975), Nicotine as the most important basic fraction of CS condensates has been verified to induce ERS (Seoane-Collazo et al., 2014; Wong et al., 2015, 2016). In this study, we showed that CS increased the expression of ERS-mediated apoptosis markers CHOP and cleaved caspase-12 in rat lungs, and nicotine increased the expression of CHOP and cleaved caspase-12 in human bronchial epithelial cells. Our previous study found serum H_2_S levels were significantly lower in smokers than nonsmokers, and were significantly lower in smokers with acute exacerbation of COPD than healthy smokers and smokers with stable COPD. Serum H_2_S levels in all patients with COPD and healthy control subjects correlated positively with pulmonary function (Chen et al., 2005). H_2_S can significantly alleviate the lung function and the lung tissue pathology score, anti-inflammation and anti-oxidation effects in COPD (Chen et al., 2011). In this study we confirmed intraperitoneal injection of NaHS in rat model of passive inhalation of CS reversed the lung tissue damage and apoptosis in lung, whereas intraperitoneal injection of endogenous H_2_S inhibitor PPG exacerbated these effects caused by CS. Intraperitoneal injection of NaHS also inhibited CS induced upregulation of ERS-mediated apoptosis markers CHOP and cleaved caspase-12. On the contrary, Intraperitoneal injection of PPG enhanced the CS induced upregulation of CHOP, and cleaved caspase-12. These results suggest that endogenous H_2_S inhibited CS induced ERS-mediated apoptosis in rat lung. *In vitro*, we also found out ERS nonspecific inhibitors taurine and 4-PBA inhibited nicotine-induced morphological changes of apoptosis in bronchial epithelial cell and upregulation of ERS-mediated apoptosis markers. The effect could be mimicked by appropriate concentration of NaHS. All these results suggest that H_2_S may inhibit bronchial epithelial cell apoptosis through regulating ERS. Recent studies have demonstrated that H_2_S inhibits ERS-mediated apoptosis in many organs. Wei et al. (2014) indicated H_2_S inhibit homocysteine-induced ERS and neuronal apoptosis in rat hippocampus. Li et al. (2015) found H_2_S protects against myocardial ischemia/reperfusion injury in rats by inhibitiing ERS. In addition, H_2_S can attenuate 6-hydroxydopamine-induced ERS in SH-SY5Y cell line (Xie et al., 2012). And H2S decreased ERS-mediated apoptosis and cardiomyocytic injury induced by hyperhomocysteinemia (Wei et al., 2010). In this study we also confirmed endogenous H_2_S can inhibit CS induced ERS-mediated apoptosis, and giving appropriate concentration of H_2_S doner inhibited nicotine induced ERS-mediated apoptosis in human bronchial epithelial cell. On the other hand, high concentrations of H_2_S have been reported to promote apoptosis of pancreatic beta-cells (Yang et al., 2007). Liu et al. (2015) also use high concentrations of H_2_S (NaHS at 56 μmol/Kg) and found out H_2_S could promote alveolar epithelial cell ERS in rats with acute lung injury. So Like NO, H_2_S may have a concentration-dependent dual effect on cell death. In this research, we found out 200 μM NaHS can inhibit nicotine-induced ERS to the greatest extent, 400 μM NaHS can not effectively inhibit nicotine-induced ERS, this result also supported the concentration-dependent dual effect on cell death of H_2_S.

ERS can activate many signal pathways, PERK-eIF2α-ATF4 signaling pathway (He et al., 2016), PI3K-Akt-NOS pathway (Alexaki et al., 2004), PERK-eIF2α-CHOP signaling (Gu et al., 2017), IRE1α-ASK1-JNK pathway (Kong et al., 2017), and IRE1α-ASK1-P38 pathway (Kong et al., 2017) have been confirmed to be involved in ERS mediated apoptosis. And Nrf2, plays an important role in oxidative stress, also involved in ERS-mediated apoptosis (Zhang et al., 2016; Wang et al., 2017). H_2_S has been confirmed to inhibit oxidative stress and inflammation, and relevant signal pathways have crosstalk with these above signals. The specific signal pathway of H_2_S on ERS-mediated apoptosis may be an important target of the protective effect of H_2_S.

Several limitations of our study must be taken into account. First, we used nicotine to induce ERS in 16HBE cells *In vitro* because it is difficult to guarantee the accuracy and consistency of the concentration of CS extract, although nicotine as the most important basic fraction of CS condensates has been verified to induce ERS (Seoane-Collazo et al., 2014; Wong et al., 2015, 2016), nicotine still cannot represent CS extract. Second, PPG only inhibits endogenous H_2_S production from cystathionine γ-lyase, maybe more H_2_S synthesis inhibitors should be used to confirm that cystathionine γ-lyase or cystathionine β-lyase play the most important role in the protective effect of H_2_S in lung. Furthermore, we used 4-PBA and taurine as the positive controls to verify the protective effect against ERS-mediated apoptosis, although taurine is effective in reducing ERS in a number of systems and taurine had been used as an ERS inhibitor in some previous studies (Lu et al., 2012; Duan et al., 2013; Liu et al., 2013; Wu et al., 2013), it did not used as a probe chemical of ERS inhibitor. Taurine maintains homeostasis of cells through antioxide activety, menbrane stability and regulating ERS. Relevant signal pathways have crosstalk with these above signals of oxidative stress and ERS, taurine can protect against ERS induced by oxidative stress (Pan et al., 2010), however, it also can inhibit oxidative stress by ameliorating ERS (Nonaka et al., 2001). In our study, we just verified H_2_S can inhibit ERS induced by nicotine, we had not detected how H_2_S inhibits ERS. Dose H_2_S directly interactions with ERS markers or indirectly regulate ERS? Thus, the specific signal pathway of H_2_S on ERS may be an important target of the protective effect of H_2_S.

In summary, this is the first study to demonstrate that H_2_S inhibited lung tissue damage by attenuating CS induced ERS in rat lung and exogenous H_2_S attenuated nicotine induced ERS-mediated apoptosis in bronchial epithelial cells. As CS is an independent risk factor for COPD and ERS is a crucial process in the pathogenesis of COPD, our findings suggest that H_2_S has potential therapeutic value in the treatment of respiratory diseases, such as COPD.

## Author contributions

FL, optimized techniques and performed the experiments and was involved in writing the manuscript. CL, YS, JZ, WL, YB, YL, ML, XN, and YH, were involved in developing some of the methods. YQ and YC, designed and guided the experiments and revised the manuscript.

### Conflict of interest statement

The authors declare that the research was conducted in the absence of any commercial or financial relationships that could be construed as a potential conflict of interest.
